# Note from the editor, January 2021

**DOI:** 10.1186/s13561-021-00311-5

**Published:** 2021-03-27

**Authors:** J.-Matthias Graf von der Schulenburg, Sebastian Braun, Jan Zeidler

**Affiliations:** 1 Leibniz University Hannover, Hannover, Germany; 2 Xcenda GmbH, Hannover, Germany; 3 Center for Health Economics Research Hannover (CHERH), Hannover, Germany

The editorial board and the editors of the open-access journal *Health Economics Review* (HER) wish all the readers a happy, healthy and blessed 2021. The turn of the year is a date to look back and develop plans for the future. Let me start with the biggest change in 2021. Due to the brilliant development of the HER, we have extended the number of editors. Sebastian Braun will now share with J.-Matthias Graf von der Schulenburg the position of Editor-in-Chief of the HER. In addition, Jan Zeidler will take over Sebastian’s position as Managing Editor. Jan Zeidler is the Managing Director of the Center for Health Economics Research Hannover (CHERH), one of the four large health economic research centers founded by the federal government of Germany. In addition, he is one of the leading experts in claims data analyses in Germany with a broad experience in health services research.

The HER was founded almost 10 years ago. Since then the development of this journal has been excellent. Our aim for the journal was to become the leading refereed open access journal in health economics, but also to develop a special focus in certain areas. We are on a good track.

The number of submissions to the journal has increased to over two-hundred per year. However, we did not decrease the quality standards and requirements: the average number of articles published per year are between 30 and 40. We plan a slight increase of the number of published articles in the years to come.

As you know, HER is open for a broad range of theoretical contributions, empirical studies and analyses of health policy with a health economic focus. Its scope includes macro- and micro economics of health care financing, health insurance and reimbursement as well as health economic evaluation, health services research, pharmacoeconomics and health policy analysis. Further research topics will be health care management and the growing importance of health care in developing countries.

Although the HER publishes on all topics in health economics, the journal is strong in a couple of topics. Many articles present research on health care issues in developing countries. The editors are very proud of the fact that the HER is strongest on developing countries in Asia and Africa among health economic journals. The editorial board plans to further strengthen the journal’s reputation on this topic. A second focus is on inequality and justice questions. However, the HER also contains articles analyzing cost-effectiveness and quality of life effects of medical interventions. We will also stimulate submissions on the economics of the treatment of rare diseases. Public authorities in the EU and North America have encouraged research on rare diseases and have improved the conditions to market innovative therapeutic concepts for rare diseases. New technologies such as the genome analysis and gene replacement therapies have improved the treatments of many orphan diseases and cancer. We would like to see more papers on the economic aspects of these issues as well as on the economics of prevention and vaccination programs.

From its start, HER-articles were listed in the most relevant databases. Our journal is listed in the *Social Sciences Citation Index*® and has an Impact Factor of 1.451 and a CiteScore of 2.9 (2019). HER articles have on the average 175.000 downloads per year, with up to 250.000 down loads.

We like to thank our colleagues in the editorial board, the authors and referees who have contributed to the HER for their support. In particular, our referees did a great job in the past. Due to the increased numbers of submissions it is getting harder to find a sufficient number of referees.

